# Comparison of a mobile phone-based malaria reporting system with source participant register data for capturing spatial and temporal trends in epidemiological indicators of malaria transmission collected by community health workers in rural Zambia

**DOI:** 10.1186/1475-2875-13-489

**Published:** 2014-12-12

**Authors:** Busiku Hamainza, Gerry F Killeen, Mulakwa Kamuliwo, Adam Bennett, Joshua O Yukich

**Affiliations:** Ministry of Health, National Malaria Control Centre, Chainama Hospital College Grounds, off Great East road, P.O. Box 32509, Lusaka, Zambia; Liverpool School of Tropical Medicine, Vector Group, Pembroke Place, Liverpool, L3 5QA UK; Biomedical & Environmental Thematic Group, Ifakara Health Institute, P.O. Box 53, Ifakara, Morogoro, United Republic of Tanzania; Malaria Elimination Initiative, Global Health Group, University of California, San Francisco, USA; Center for Applied Malaria Research and Evaluation (CAMRE), Tulane University School of Public Health and Tropical Medicine, New Orleans, LA USA

**Keywords:** Community health worker, Malaria infections, Diagnostic positivity, Surveillance, Short messaging system, Patient register

## Abstract

**Background:**

Timeliness, completeness, and accuracy are key requirements for any surveillance system to reliably monitor disease burden and guide efficient resource prioritization. Evidence that electronic reporting of malaria cases by community health workers (CHWs) meet these requirements remains limited.

**Methodology:**

Residents of two adjacent rural districts in Zambia were provided with both passive and active malaria testing and treatment services with malaria rapid diagnostic tests (RDTs) and artemisinin-based combination therapy by 42 CHWs serving 14 population clusters centred around public sector health facilities. Reference data describing total numbers of RDT-detected infections and diagnostic positivity (DP) were extracted from detailed participant register books kept by CHWs. These were compared with equivalent weekly summaries relayed directly by the CHWs themselves through a mobile phone short messaging system (SMS) reporting platform.

**Results:**

Slightly more RDT-detected malaria infections were recorded in extracted participant registers than were reported in weekly mobile phone summaries but the difference was equivalent to only 19.2% (31,665 *versus* 25,583, respectively). The majority (81%) of weekly SMS reports were received within one week and the remainder within one month. Overall mean [95% confidence limits] difference between the numbers of register-recorded and SMS-reported RDT-detected malaria infections per CHW per week, as estimated by the Bland Altman method, was only −2.3 [−21.9, 17.2]. The mean [range] for both the number of RDT-detected malaria infections (86 [0, 463] *versus* 73.6 [0, 519], respectively)) and DP (22.8% [0.0 to 96.3%] *versus* 23.2% [0.4 to 75.8%], respectively) reported by SMS were generally very consistent with those recorded in the reference paper-based register data and exhibited similar seasonality patterns across all study clusters. Overall, mean relative differences in the SMS reports and reference register data were more consistent with each other for DP than for absolute numbers of RDT-detected infections, presumably because this indicator is robust to variations in patient reporting rates by location, weather, season and calendar event because these are included in both the nominator and denominator.

**Discussion/Conclusion:**

The SMS reports captured malaria transmission trends with adequate accuracy and could be used for population-wide, continuous, longitudinal monitoring of malaria transmission.

## Background

Like many malaria endemic countries, Zambia is experiencing an epidemiological transition with regard to malaria disease burden, with a notable national decline in parasite prevalence and incidence [1]. Public health surveillance, has been defined as the "ongoing systematic collection, analysis, and interpretation of data critical to the planning, implementation, and evaluation of public health interventions” [2, 3]. Effective use of such surveillance data requires timely dissemination to all relevant stakeholders [2, 3]. Effective systems for detecting and reporting malaria infection in human populations have an increasingly important role to play as control steadily progresses towards elimination so that infection and disease become more focal in time and space and additional interventions are increasingly targeted in response to surveillance data [4–6].

A viable surveillance system for malaria in Zambia needs to routinely collect sufficient data to describe the population’s health status [7], for the purpose of detecting temporal and spatial variations in epidemiological profile, including those arising directly from changes in clinical and public health practices. In Zambia, the standard national surveillance system is the integrated health management information system (HMIS) [8]. Currently the HMIS is operational at all established health facilities in the country and requires monthly reporting from all medical office teams that aggregate and summarize these facility reports. Currently, there are 22 malaria-specific indicators reported through the HMIS which encompass disease burden, as well as use and availability of commodities for prevention, diagnosis and treatment [8]. As in most countries in sub-Saharan Africa, the completeness, accuracy and timeliness of HMIS in Zambia are often inadequate or, at the very least, questionable. These systematic weaknesses undermine stakeholder confidence in the reliability of this data and, consequently lead to its under-utilization for decision-making and planning [9].

In poorly-resourced countries, community-based surveillance systems (CBSS) can complement health facility (HF)-based surveillance. This is of particular relevance to malaria control because the infectious human reservoir is primarily comprised of chronic infections associated with sub-acute symptoms distributed across entire at-risk populations [10] and both rapid diagnostic tests (RDTs) and artemisinin-based combination therapies (ACTs) for uncomplicated malaria can be utilised appropriately by community health workers (CHWs) with minimal general education and technical training [11–16]. Additionally, CHWs are closer to the community as they reside in the communities they provide service to, thus increasing access and potentially capturing more fever cases experienced within the community [11, 17, 18]. CBSS can provide quantitative estimates of disease burden in a defined population and service delivery indicators for disease control measures [19] but remain under-exploited in relation to malaria, with only 12 million RDTs being used by CHWs globally, 11 million of which are accounted for by India alone [20]. Several studies have demonstrated the ability of CHWs to collect epidemiological data on a variety of diseases, including malaria, provided they have appropriate training and can relay their observations through appropriately tailored reporting systems [10, 13, 15, 21–23]. Data from CBSS can be utilised to describe spatial and temporal patterns of variation in infection or disease incidence, as well as access to and use of preventive services, so that interventions and resources can be rationally allocated at fine scales across large-scale programmes [5, 10, 24]. While there is widespread consensus on the benefits of electronic reporting systems for relaying and collating data from CBSS in programmatic contexts [25–27], evidence to support this view remains limited to remarkably few studies [27–29].

This study describes and evaluates a prototype mobile phone reporting platform for a CBSS in rural Zambia that was initially established as a programme implemented by CHWs for community-wide passive and active testing with RDTs and treatment of all confirmed cases with Artemether-Lumefantrine (AL), which also allowed monitoring of malaria parasite infection burden as a secondary objective [10].

## Methods

### Study sites

**Figure 1:**
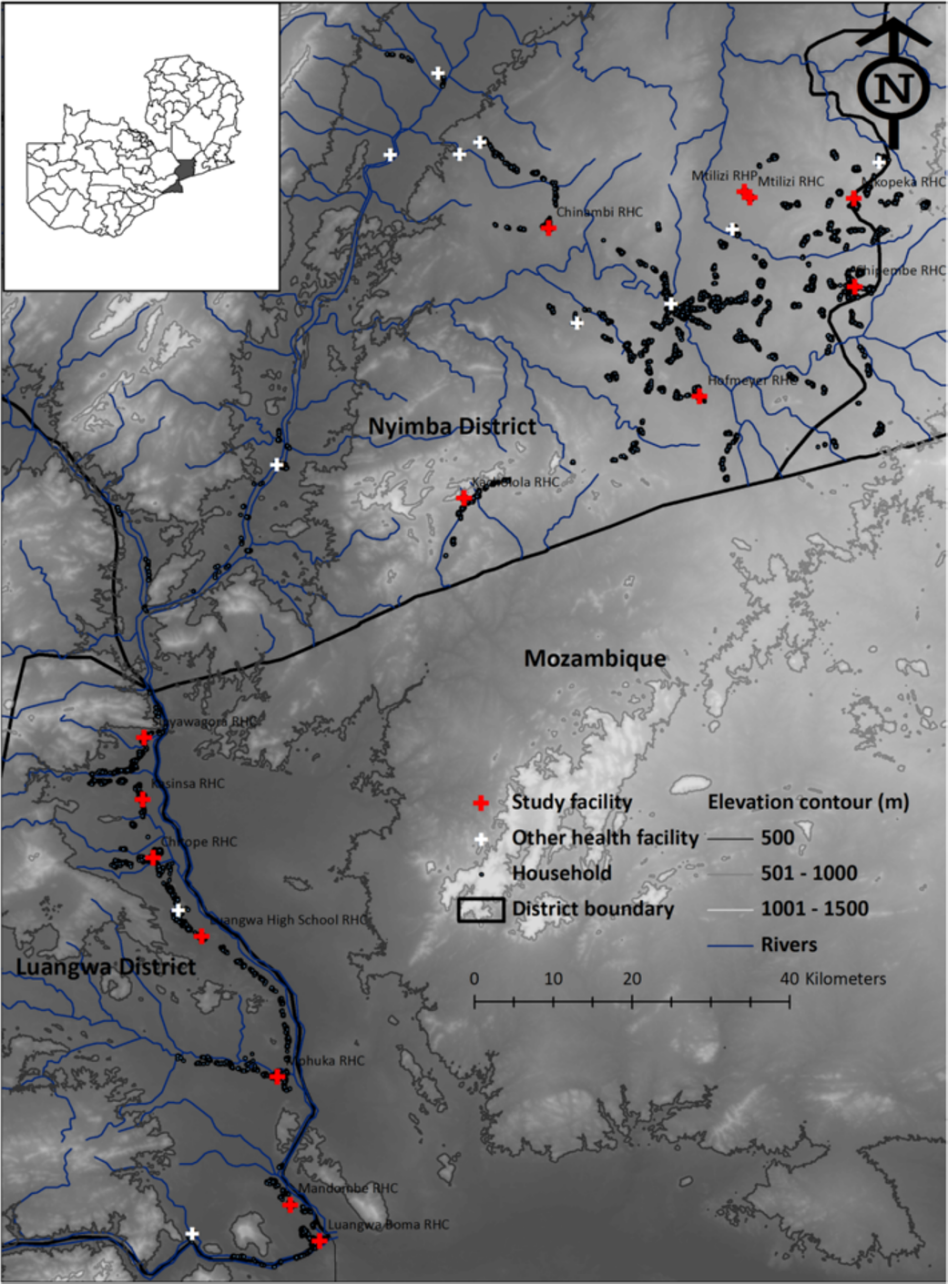
**Map showing the selected health facilities around which the community health workers, and the catchment populations they served, were located.**

A CBSS was established in the rural districts of Luangwa and Nyimba, respectively located in Lusaka and Eastern provinces of Zambia (Figure 1) [10]. In these districts, perennial, intense transmission of *Plasmodium falciparum* is mediated by *Anopheles funestus* Giles at an estimated entomological inoculation rate of approximately 70 infectious bites per unprotected person per year [30]. Luangwa (3,468 km^2^) is located 325 kilometres south-east of Lusaka, the capital city of Zambia. The district’s total population is approximately 27,560 residents and it has an annual estimated population growth rate of 2.9% [31]. Nyimba is a larger district (10,943 km^2^), located 350 kilometres east of Lusaka, with an approximate population of 108,637 inhabitants and an estimated annual population growth rate of 3.4% [31]. Both districts have similar annual trends in mean malaria diagnostic positivity with Nyimba having a higher maxima (31.0%) than that of Luangwa (17.2%) [10].

### Testing and treatment

As described in detail elsewhere [10], 14 population clusters (7 in each district), consisting of the nearest consenting 165 households to a selected public sector HF were selected and enrolled to participate in longitudinal parasite surveys conducted by paid CHWs. Each cluster had 3 CHWs, resulting in a total of 42 CHWs, and thus a corresponding number of reporting units, distributed across the study area. Parasitological assessments were conducted continuously from January 2011 to March 2013 in Luangwa and from April 2011 to March 2013 in Nyimba district in all the selected clusters. All consenting households received monthly active visits from CHWs, which included parasitological surveys using RDTs detecting histidine-rich protein 2 antigen (Malaria Pf cassette test, ICT Diagnostics), coupled with registers designed in a pre-defined questionnaire format [10]. Consent for household participation was given by the head of the household and consent was obtained from individual study participants, or parents/guardians in the case of minors, for the RDT test. If they developed any symptoms in between these active visits, study participants were encouraged to seek care through passively-offered diagnosis and treatment services, either from the CHWs at their place of residence or at the nearest HF. All participants found positive for malaria infection were treated with AL as per national policy [32]. All participants encountered in either the active or passive visits that were found to be negative for malaria infection, but were febrile or complained of any other symptom of illness, were referred to the nearest HF.

### Questionnaire surveys

The CHWs’ primary method of collecting study participant information was through the use of a paper-based, pre-defined questionnaire provided in the physical form of a register book. A total of 16 data elements were captured through this method and these included date, cluster, participant identification number, sex, age, visit type (active/passive), village, axilliary temperature, RDT results, clinical symptoms of illness (fever, history of fever, headache, cough, diarrhoea, vomiting, breathing problems, chest pain and any other symptoms), treatment provided and participant outcome, in addition to access and utilization of specific preventive measures, namely long-lasting insecticidal nets (LLINs), indoor residual spraying (IRS) and intermittent preventive therapy. All the entries in the registers, with each line of data corresponding to a single participant contact, were made in duplicate with carbon paper. During the monthly supervisory visits, original copies of these reports were collected and transported to the NMCC for entry into a correspondingly structured Microsoft excel spreadsheet in fully explicit, line-listed electronic database format, while the CHWs retained the duplicate copies for their own records. All data from the registers were double-entered and verified, reconciled, and then cleaned following descriptive frequency analysis of the distributions of values for each variable.

### Weekly summary reporting of aggregate data via short message service

**Figure 2:**
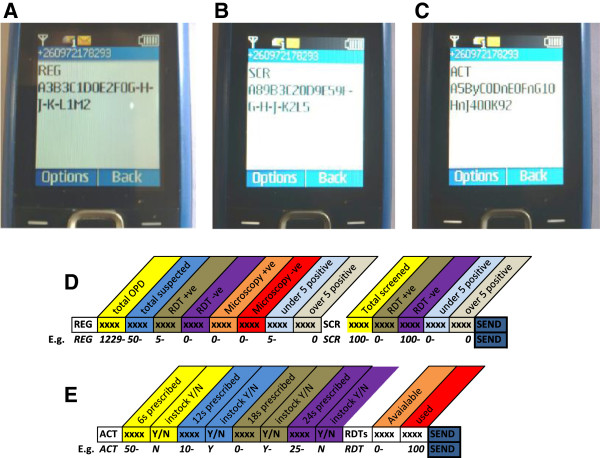
**Screenshot examples of Community Health Worker (CHW) mobile phone short messaging service (SMS) text before transmission to the National Malaria Control Centre and an Illustration of the interpretation of the code sent through the SMS reporting system. A**: example of an SMS code for study participants that were attended to through the passive system when they self-reported to the CHW, **B**: example of an SMS code for study participants contacted through active monthly household visits, **C**: Example of an SMS code for reporting on use and availability of AL and RDTs, **D**: Schematic Illustration of the format and syntax of reports for diagnostic results from both passive and active visits, and **E**: Schematic Illustration of the format and syntax of reports for the availability of AL and RDTs. REG refers to passive participants data, SCR refers to active participant data, ACT refers to Artemether Lumefantrine stocks, RDT refers to rapid diagnostic test stocks, OPD refers to passive participant contact, and Y or N – refer to stock availability (Yes or No). Note that CHWs did not report fields H, J, K in the REG report or G, H, J in the SCR report because these represent microscopy data elements that were only collected by the health facilities.

In addition to the paper-based participant registers, weekly aggregate summary reports of these data were collated through mobile phone short messaging service (SMS) via the Airtel® network (Figure 2). A paper-based form summarizing the aggregated data elements to be transmitted via SMS was completed by each CHW each week, based on the fully explicit data recorded in the participant register books. These weekly summaries were then transmitted to a mobile phone at the NMCC via SMS text in a pre-determined format, every Friday (Figure 2). A total of 25 data elements were transmitted through this method, which included the total number of participants in both active and passive visits, number of positive and negative cases disaggregated by age and type of visit, number with and without fever, in addition to summaries of various AL pack sizes dispensed and remaining, and similar indicators for the use and availability of RDTs (Table 1). Once each data message was received by the NMCC, an acknowledgement message was manually sent back to the sender by a data entry associate. The data was reviewed by the study team and the CHWs were contacted by voice via mobile phone for verification or correction of the data if any clarifications were required. If there was need to correct the data the CHWs would not be required to resend the data but would instead be requested during the call to change their paper versions of the SMS summary to reflect the corrected data for their records. The study team would then correspondingly also change the relevant data entries in the database. Once the verification process was complete, the data were then compiled into a Microsoft Excel® spreadsheet in database format. The mobile phones were also used for communication among the CHWs and staff at their supervising health facility. In addition to provision of a basic mobile phone at a cost of ZMW 50 (approximately US$9.47 based on the 2012 average annual exchange rate of ZMW 5.23 to US$1 [33]) for each CHW, a monthly air time top-up worth ZMW20 (US$3.32) was electronically sent to each CHW in order to facilitate transmission of the SMS reports and any other communication requirements.

**Table 1:** **Data element definitions for all data transmitted through the SMS mobile reporting system**

Data element	Definition
REG (A)TOTAL OPD	Total number of study participants that sort care from the CHWS
REG (B) TOTAL SUSPECTED	Total number of study participants that sort care from the CHWs and were suspected of having malaria
REG (C) UNDER 5 RDT +VE	Total number of study participants that sort care from CHWs under the age of five and had a positive rapid diagnostic test result
REG (D) UNDER 5 RDT -VE	Total number of study participants that sort care from CHWs under the age of five and had a negative rapid diagnostic test result
REG (E) OVER 5 RDT +VE	Total number of study participants that sort care from CHWs over the age of five and had a positive rapid diagnostic test result
REG (F) OVER 5 RDT -VE	Total number of study participants that sort care from CHWs over the age of five and had a negative rapid diagnostic test result
REG (L) FEVER UNDER 5	Total number of study participants that sort care from CHWs under the age of five who were febrile
REG (M) FEVER OVER 5	Total number of study participants that sort care from CHWs over the age of five who were febrile
SCR (A) TOTAL SCREENED	Total number of study participants that consented to actively being tested for malaria by CHWs
SCR (B) UNDER 5 +VE	Total number of study participants that consented to actively being tested for malaria by CHWs under the age of five and had a positive rapid diagnostic test result
SCR (C) UNDER 5 RDT -VE	Total number of study participants that consented to actively being tested for malaria by CHWs under the age of five and had a negative rapid diagnostic test result
SCR (D) OVER 5 RDT +VE	Total number of study participants that consented to actively being tested for malaria by CHWs over the age of five and had a positive rapid diagnostic test result
SCR (E) OVER 5 RDT -VE	Total number of study participants that consented to actively being tested for malaria by CHWs over the age of five and had a negative rapid diagnostic test result
SCR (K) FEVER UNDER 5	Total number of study participants that consented to actively being tested for malaria by CHWs under the age of five who were febrile
SCR (L) FEVER OVER 5	Total number of study participants that consented to actively being tested for malaria by CHWs over the age of five who were febrile
(A) 6S PRESCRIBED	Total number of AL 6 packs prescribed and provided to study participants
(B) INSTOCK	Total available AL 6 packs
(C) 12S PRESCRIBED	Total number of AL 12 packs prescribed and provided to study participants
(D) INSTOCK	Total available AL 12 packs
(E) 18S PRESCRIBED	Total number of AL 18 packs prescribed and provided to study participants
(F) INSTOCK	Total available AL 18 packs
(G) 24S PRESCRIBED	Total number of AL 24 packs prescribed and provided to study participants
(H) INSTOCK	Total available AL 24 packs
(J) RDT AVAILABLE	Total available individual rapid diagnostic tests
(K) RDT USED	Total used individual rapid diagnostic tests

### Concordance evaluation

The explicit, unaggregated, line-listed, manually double-entered reference data from the participant registers were independently aggregated by the NMCC into weekly estimates of total number of malaria infections seen by each CHW and the corresponding diagnostic positivity (DP) of tests associated with those participant contacts. The weekly DP was estimated as outlined in the formula below:


The strength of association of the overall number of malaria infections recorded in the reference register data with those reported via SMS was expressed in terms of the correlation coefficient (*r*) between the two data capture methods, which were also disaggregated by type of patient contact. Though one limitation of analysis by correlation coefficient is that it is possible to obtain high correlation even when the absolute values of the variables are different [34, 35]. Thus in order to estimate the degree of agreement of these data sets, the numbers of infections detected by each CHW each week were subjected to Bland-Altman statistical analysis, which is a graphical analysis method whereby the difference between the weekly total number of study participant contacts from each reporting system was plotted against the mean across the two reporting systems to allow for measurement of the magnitude of the errors, as well as the trends in magnitude of the errors, relative to the magnitude of the total number of malaria infections recorded in the patient registers and reported through the SMS reporting system [36]. Time trend analysis was also applied to the weekly SMS reports compared to the reference register data summaries of weekly total number of malaria infections data and DP in order to further assess the correlation between these datasets as a function of time lag. The total number of malaria infections time series data were further analysed by cluster using a bivariate local polynomial regression, specifically the locally-weighted scatter-plot smoother (LOESS) to provide a visually smoothened fit of time series from each of the two reporting systems by identification of, and minimization of influence, of possible outlier data [37, 38]. Additionally this analysis also provided a qualitative comparison of the mean relative difference, both overall and disaggregated by type of participant contact, as well as time trends and seasonality of data provided from the reference paper-based records *versus* those reported in the SMS system.

### Ethical approval

Ethical approval was obtained from the University of Zambia, Biomedical Research Ethics Committee (Reference 004-05-09) and the Research Ethics Committee of the Liverpool School of Tropical Medicine (Approval 09.60). Authority to conduct the study was also obtained from the Ministry of Health in Lusaka, Zambia.

## Results

The reference paper-based register recorded an overall total of 129 weeks of data, while the SMS reporting system reported only 120 weeks of data, due to delayed initiation and earlier termination of the latter when funding ran out towards the end of the study. Out of an expected 5040 SMS weekly summaries, only 2934 (58.2%) were submitted by the CHWs. This may have been due to the lack of power sources for charging the mobile phones because most of the study sites were not connected to the national power grid. This resulted in the CHWs travelling long distances to charge their phones. Additionally this may also have been due to unstable mobile phone network coverage particularly in the most rural of the study sites. Of the 2934 SMS weekly summaries submitted by the CHWs, 81% (2375/2934) were submitted according to the timeliness targets at the outset (weekly) and the remainder (19% (559/2934)) within one month. These delays in reporting a minority of weekly summaries may have been due to a lack of access to mobile phone network coverage, particularly in the most remote parts of the study area. However, CHWs were encouraged to transmit any missing reports whenever they had access to mobile phone coverage. In some limited situations, this lead to a few CHWs choosing to transmit all their data at the end of the month when they travelled to areas of good mobile phone coverage, such as when they visited the HFs to collect supplies and submit the reference paper-based register records. All analyses of concordance reported here were based only on comparisons of data summaries from the reference paper-based register records and SMS reports where both were available for the same CHW in the same week, which ranged from 40 to 100 weeks per CHW. All detected malaria infections considered in the analyses of these data refer only to infections, which were detected parasitologically by RDT, rather than by clinical symptoms alone.

An overall total of 31,665 and 25,583 detected malaria infections were recorded through the paper-based participant register and reported through the SMS platform, respectively. The mean weekly number of RDT-detected malaria infections recorded by each CHW in the reference paper-based register was 7.1 (1.1 and 6.0 from the passive and active participant contacts, respectively), while that reported in the SMS systems was 9.0 (2.3 and 6.7 from passive and active contacts, respectively).

There was a clear association between the overall number of RDT-detected malaria infections recorded by the reference paper-based register and reported by SMS platform (r [95% CI] = 0.64 [0.61, 0.66], p < 0.001). This association was also clear when the data were disaggregated by participant contact type, with a stronger correlation for the active contacts (r [95% CI] = 0.68 [0.65, 0.69], p < 0.001) than the passive contacts (r [95%CI] = 0.36 [0.33, 0.39], p < 0.001), as evidenced by the higher correlation coefficient and the lack of overlap between the confidence intervals for the two participant contact types.

**Figure 3:**
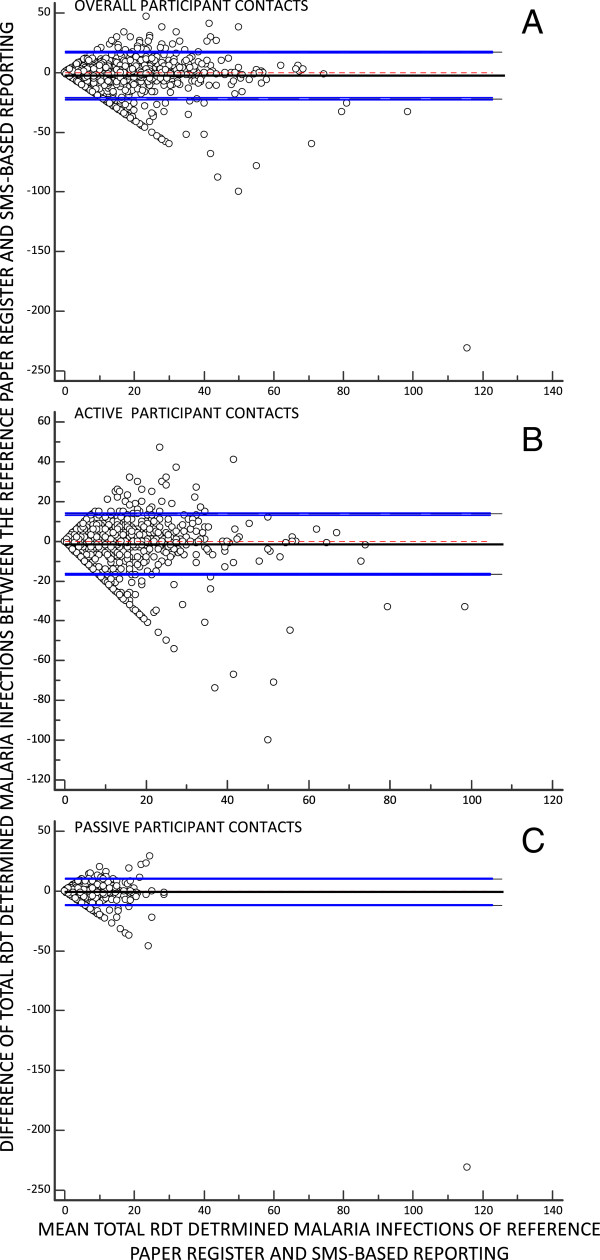
**Bland-Altman plots of the difference between weekly numbers of rapid diagnostic test detected malaria infections recorded in the reference paper register and reported by SMS against their mean, overall (A), or disaggregated into active (B) and passive (C) participant contacts.**

The overall mean [95% limits of agreement (LOA)] difference in the number of RDT-detected malaria infections per week per CHW between the reference paper-based register and SMS reporting platform, as estimated by the Bland-Altman method, was −2.3 ([−21.9, 17.2]) infections per CHW per week (Figure 3). Bland-Altman estimates for the mean [95% LOA] difference in the number of register-recorded versus SMS-reported malaria infections in active participant contacts was −1.4 ([−16.5, 13.7]) infections per CHW per week and less for passive participant contacts at −0.9 ([−12.0 to 10.1]) infections per CHW per week (Figure 3). Thus, observations on the numbers of actively and passively-detected malaria infections per CHW per week, as well as their combined total are approximately similar. Nevertheless, there is a clear need for improved quality control based on the considerable amount of data discordance reflected in the wide 95% LOA that suggests some degree of caution should be exercises when interpreting these data. While the DP of participants tested has proven a robust indicator of malaria risk as recorded in the reference patient registers [10], the binomial distribution that presumably stabilizes this proportional outcome to variations in participant contact rates also precludes the use of Bland-Altman analysis. However, it was possible to compare trends in the weekly aggregate values for RDT-detected malaria infections and DP outcomes descriptively and using LOESS regression

**Figure 4:**
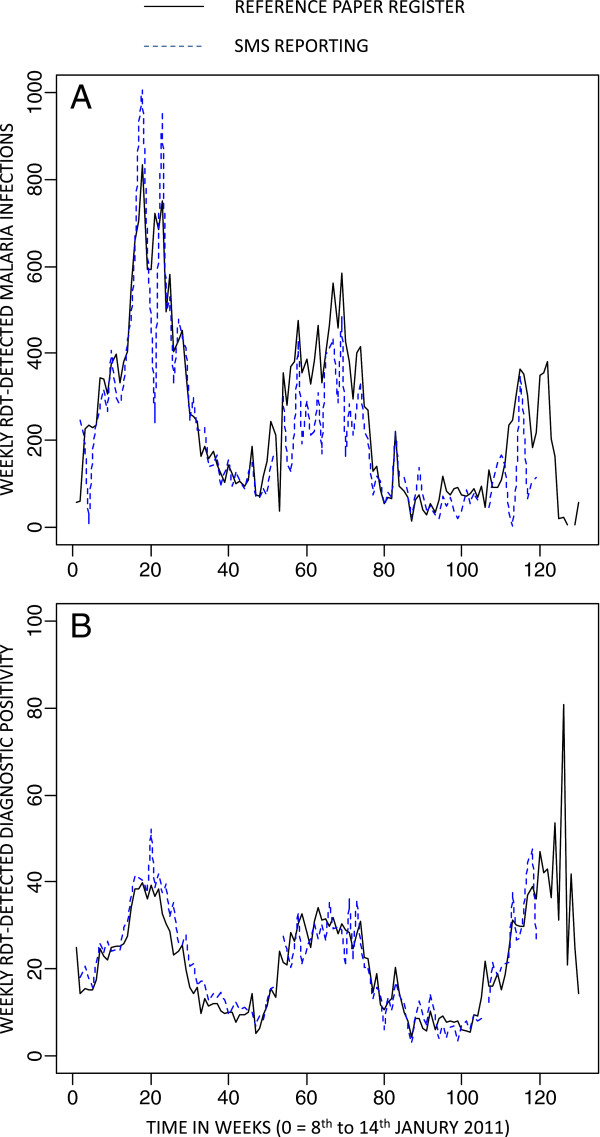
**Time trend series plot of weekly means, aggregated across all community health workers, for number of rapid diagnostic test detected malaria infections (A) and associated diagnostic positivity (B) as recorded by the reference paper-based registers and reported by SMS.**

**Figure 5:**
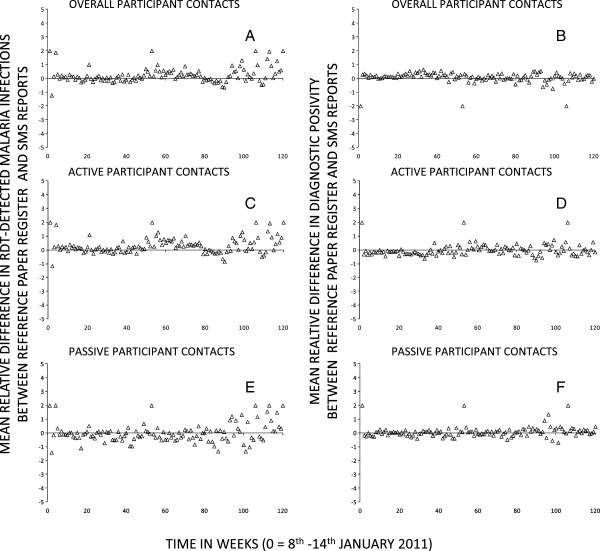
**Difference plots for number of rapid diagnostic test detected malaria infections (A, C, E) and diagnostic positivity (B, D, F) as recorded by the reference paper register and reported by SMS overall (A and B), or disaggregated by active (C and D) and passive (E and F) participant contacts.**

.Trends over time for the overall number of malaria infections (Figure 4A) and, even more so, overall DP (Figure 4B) aggregated by week across all CHWs suggests a close positive association between the paper-based reference register and SMS reporting system. On average, the two datasets closely matched each other in absolute terms and exhibited very similar temporal variations that captured comparable seasonal trends, with both maxima and minima consistently occurring at approximately the same times. This observation was confirmed when these two malaria infection burden indicators were compared in terms of their mean relative difference overall (Figure 5A and B), and when disaggregated into active (Figure 5C and D) and passive (Figure 5E and F) participant contacts. Overall, mean relative differences in the SMS reports and reference register data were more consistent with each other for DP than for absolute numbers of RDT-detected infections, presumably because DP is robust to variations in patient reporting rates by location, weather, season and calendar event because these are included in both the nominator and denominator.

**Figure 6:**
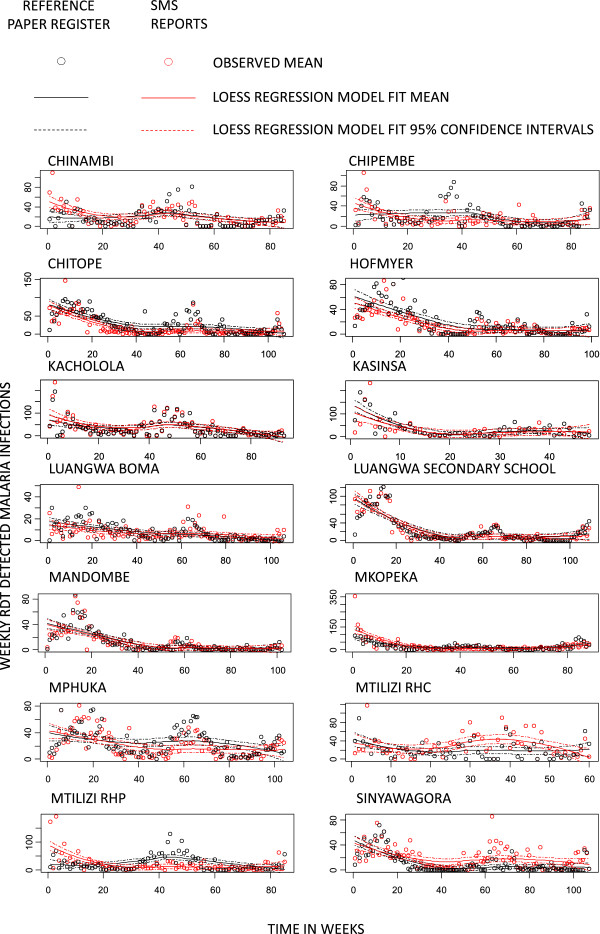
**Locally weighted scatter-plot smoother (LOESS) plots of weekly cluster-disaggregated mean number of rapid diagnostic test detected malaria infections recorded by the reference paper-based registers and reported in the SMS system against time in weeks of reporting.**

**Figure 7:**
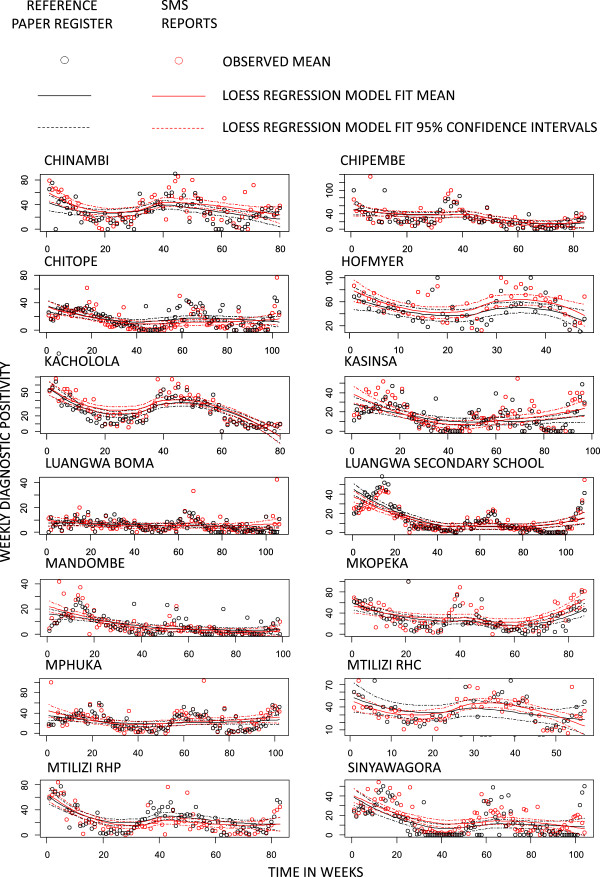
**Locally weighted scatter-plot smoother (LOESS) plots of weekly cluster-disaggregated diagnostic positivity recorded by the reference paper-based registers and reported in the SMS system against time in weeks of reporting.**

Cluster-disaggregated time series for both the reference paper registers and SMS reports also exhibited similar time trends and seasonal variation in the number of RDT-detected malaria infections (Figure 6) and especially DP (Figure 7). Visual inspection of the results of LOESS regression, using identical bandwidths within each cluster, indicate that the SMS reporting system captures similar temporal and geographic variation to that recorded in the reference data detailed in the participant register.

A comprehensive cost analysis of the full running costs of CBSS, exclusive of SMS reporting system and costs, is described in detail elsewhere [10]. However at a total annual provider fee running cost of only US$39.84 and a single expenditure of US$9.47 for the mobile phone per CHW at start up, this SMS reporting system may be considered an affordable, inexpensive operational supplement to this CBSS that may dramatically enhance the *de facto* value of its data recording and reporting mechanisms.

## Discussion

The CBSS captures large volumes of data on population characteristics (age, sex and residence), symptomology, morbidity, diagnostic testing and treatment for malaria, as well as LLINs and IRS, which are not routinely captured by the national HMIS [8, 10]. This study demonstrated that CHWs are able to reliably record, summarize and transmit these data through an electronic reporting platform, in this case a mobile phone.

The comparison of the SMS weekly summaries with the reference paper-based, quality-controlled register records indicates some modest incompleteness of the former, with the paper based system recording a higher overall number of malaria infections. This may have been due to under-reporting in the SMS reporting system resulting from challenges in accurately summarising the reference registers data and non-submission of the SMS reports by some of the CHWs when they were required to. Additionally, the manual entry of the SMS-transmitted data into an excel spreadsheet by the study team may have resulted in some reports being lost in the process and not entered into the database because, unlike the paper-based register records, there was no double entry verification of this data.

Timeliness, a key performance measure of any public health reporting system [39, 40] was achieved by the SMS based platform in most instances, as these reports were received as expected within a week in the vast majority of cases, and within a reasonably adequate timeline of one month the rest of the time. Monthly quality control visits to CHWs were primarily provided to support correct recording of data in the reference data on paper, in the form of a detailed review of the data collected when the registers were handed over to the supervision team. During these visits the SMS reporting records were also reviewed but in most cases the CHWs had already submitted at least some of their SMS reports prior to the visit.

In the reference paper-based register, the high level of detail that included individual-level data over the entire implementation period provided an invaluable resource in the form of a rigorous epidemiological database for critical analysis of malaria transmission dynamics and risk factors [10]. While such comprehensive, detailed data entry and analysis is not realistically feasible beyond the unusually well-resourced context of such a research study, summaries of these indicators could nevertheless be included in scalable electronic reporting systems similar to the one described here. These data could support planning processes at district, provincial and national levels, and also assess independent reports from these levels on needs, forecasts, access and use of various malaria interventions. Additionally, these data may be used for targeting interventions as malaria transmission drops as these SMS based reports demonstrate adequacy in reflecting epidemiological trends in a population on a timely basis. Although not explicitly explored in this study, the findings suggest a role for SMS based reporting as a potential management tool to monitor performance of frontline health workers, in this case the CHWs. This was exemplified in this study through the monitoring of CHWs performance as to whether they achieved the targeted number of active household visits per week and tracking the availability and use of AL and RDTs.

An obvious limitation of the study was that the SMS platform would have benefited from an automated central server system of data capture when SMS reports were submitted [41], which would have reduced human error in the form of missing or duplicated data and transcription errors.

## Conclusion

This SMS reporting system captured malaria transmission trends, as recorded in the reference paper based registers, with adequate accuracy, suggesting potential use in population-wide, continuous and longitudinal monitoring of temporal and geographic trends in disease incidence. Additionally, the summary reports through the SMS platform enabled reasonably timely collation, access to and dissemination of the paper-based surveillance data, which was shared among the study team and district health management teams so they could be used for programmatic decision-making. Given the recent dramatic growth in mobile phone networks globally, and in Africa particularly, such CBSS may be highly effective if mobile technology platforms are harnessed for reporting directly from CHWs to central servers to allow for rapid data access, use and quality assurance by stakeholders at the district, provincial and national levels.

## Abbreviations

AL: Artemether Lumefantrine
CBSS: Community based surveillance system
CHW: Community health worker
DP: Diagnostic positivity
HF: Health facility
RDT: Rapid diagnostic tests
SMS: Short messaging system
LLINs: Long-lasting insecticidal nets
LOESS: Locally weighted scatter-plot smoother
IRS: Indoor residual spraying
NMCC: National Malaria Control Centre.
